# A novel digital rectoscope for the triage of lower gastrointestinal symptoms in primary care: a prospective multicentre feasibility study

**DOI:** 10.3399/BJGPO.2022.0036

**Published:** 2022-08-24

**Authors:** James Lewis, Alan Askari, Arihant Mehta, Yasmin Razak, Prash Patel, Ravi Misra, Henry Tilney, Tanveer Ahmed, Mooyad Ahmed, Adnan Syeed, John Camilleri-Brennan, Ralph John Nicholls, James Macalister Kinross

**Affiliations:** 1 Clinical Research Fellow, Imperial College Healthcare NHS Trust, London, UK; 2 Clinical Research Fellow, St Mark’s Hospital, London, UK; 3 Medical Student, School of Medicine, King’s College London, London, UK; 4 GP, The Golborne Medical Centre, London, UK; 5 GP, Magnolia House Practice, Ascot, UK; 6 Consultant Gastroenterologist, St Mark’s Hospital, London, UK; 7 Consultant Colorectal and Robotic Surgeon, Frimley Park Hospital, London, UK; 8 GP, Shifa Surgery, Blackburn, UK; 9 Consultant General Surgeon, East Lancashire Hospitals NHS Trust, London, UK; 10 GP, Forth Medical Group, Forth Valley, UK; 11 Consultant General and Colorectal Surgeon, Forth Valley Royal Hospital, Forth Valley, UK; 12 Visiting Professor in Colorectal Surgery, Department of Biosurgery and Surgical Technology, Imperial College London, London, UK; 13 Consultant Colorectal Surgeon, Imperial College Healthcare NHS Trust, London, UK; 14 Senior Lecturer in Colorectal Surgery, Faculty of Medicine, Department of Surgery & Cancer, Imperial College London, London, UK

**Keywords:** rectoscopy, general practice, family practice, colorectal, endoscopy, COVID-19

## Abstract

**Background:**

Access to community rectoscopy might help to ease the burden on hospital services and reduce costs for the NHS. To assess this, a prospective multicentre observational phase I feasibility study of a novel digital rectoscope and telestration software for the triage of lower gastrointestinal (GI) symptoms was undertaken.

**Aim:**

To determine if digital rectoscopy is feasible, acceptable, and clinically safe.

**Design & setting:**

Evaluation of clinician case reports and patient questionnaires from patients recruited from five primary care centres.

**Method:**

Adults meeting 2-week wait (2WW) criteria for suspected lower GI cancer, suspected new diagnosis, or flare-up of inflammatory bowel disease (IBD) were enrolled. Examinations were performed by primary care practitioners using the LumenEye rectoscope. The CHiP platform allowed immediate remote review by secondary care. A prospective analysis was performed of patient and clinician experiences, diagnostic accuracy, and cost.

**Results:**

A total of 114 patients were recruited and 110 underwent the procedure (46 [42%] females and 64 [58%] males). No serious adverse events were reported. Eighty-two (74.5%) patients reported that examination was more comfortable than expected, while 104 (94.5%) felt the intervention was most convenient if delivered in the community. Clinicians were confident of their assessment in 100 (87.7%) examinations. Forty-eight (42.1%) patients subsequently underwent colonoscopy, flexible sigmoidoscopy, or computed tomography virtual colonoscopy (CTVC). The overall sensitivity and specificity of LumenEye in identifying rectal pathology was 90.0% and 88.9%. It was 100% and 100% for cancer, and 83.3% and 97.8% for polyps. Following LumenEye examination, 19 (17.3%) patients were discharged, with projected savings of 11 305 GBP.

**Conclusion:**

Digital rectoscopy in primary care is safe, acceptable, and can reduce referrals. A phase III randomised controlled trial is indicated to define its utility in reducing the burden on hospital diagnostic services.

## How this fits in

Pressures on endoscopy services are unsustainable. LumenEye is a digital rectoscope coupled to a telestration software platform, which enables rectal visualisation to be performed with access to a real-time remote second opinion, which is safe and acceptable to both patients and clinicians. This study will be used to power a phase III trial aimed to reassess NHS lower GI 2WW criteria.

## Introduction

Each year 20% of patients attending primary care clinics in the NHS report having experienced rectal bleeding.^1^ Rectal bleeding is most frequently caused by benign conditions of the anorectum that can be safely managed in the community. Rectoscopy in primary care is currently limited to a small number of practices because of training limitations, lack of equipment, and prohibitive costs.^2^ If the technique was more widely available, benign and malignant rectal lesions could be more appropriately stratified along appropriate referral pathways, and the need for referral altogether could be avoided in some cases. In the 2019–2020 financial year, 441 689 patients were referred to secondary care in England with suspected lower GI cancer, based on the 2WW criteria.^3^ In more than one-third, where a diagnosis of colorectal cancer was made, the tumour was located in the rectum.^4^ Access to community rectoscopy would result in a reduction of the presently unsustainable burden on hospital flexible endoscopy, which has been exacerbated by the COVID-19 pandemic, and reduce NHS costs.^5–10^

**Figure 1.**
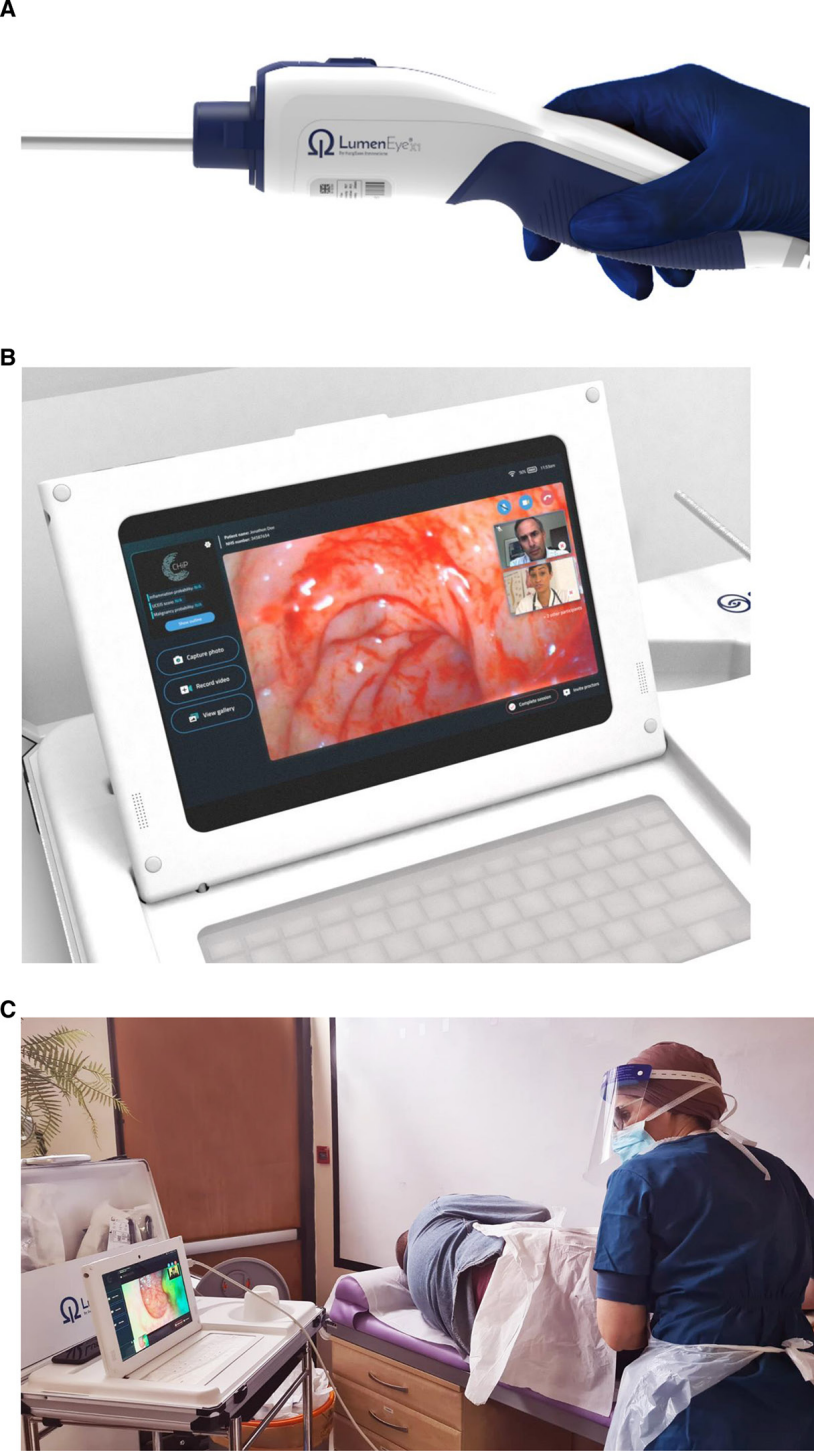
The LumenEye system A - The LumenEye® device without its disposable plastic sleeve. The disposable plastic sleeve is graduated to 15cm and includes a 3mm working channel to accommodate endoscopic instruments. The handle contains an air insufflator bellows (blue) allowing single handed use. B - The LumenEye® dock with the CHiP software. A typical view during a live specialist ‘proctoring’ examination with webcam displays of both the examining clinician and proctor projected over the view from the rectoscope. C - The LumenEye® in a typical deployment in a GP practice with remote secondary care review via CHiP.

A rectoscope potentially suitable for use in primary care in patients presenting with rectal bleeding has been developed.^11^ The LumenEye X1 (SurgEase Innovations, London, UK) (Figure 1A) is a low-cost device that allows still or video-rectal images, taken during examination in primary care, to be transmitted via a Wi-Fi link through its software (‘CHiP’, Figure 1B and C ) to a clinician in secondary care, enabling real-time assessment of pathology and a decision on the need for referral.

A phase I study was carried out to determine the feasibility and clinician and patient acceptability of the use of the LumenEye in primary care. Further data on safety and diagnostic accuracy were also obtained.

## Method

### Study design and setting

LuCID is a prospective, multicentre observational feasibility study of a novel digital endo-rectal examination device called the LumenEye X1 in primary care in patients with lower GI symptoms fulfilling the criteria for a 2WW pathway referral^12^ or IBD.

The study sites were NHS primary care practices located in Scotland (Forth Valley) and England (Blackburn, London [north and south], and Berkshire). Primary care practitioners were invited to take part in the study by letter of invitation. Endoscopic examinations were performed in a primary care setting by clinicians who had completed training on device usage, but not disease recognition. Colorectal surgeons delivered training using a synthetic simulated rectal model followed by at least five observed procedures. Training competency included the following: 1) safe operation of the device with appropriate sterilisation; 2) reproducible identification of key anatomical landmarks; and 3) quality assurance assessment including bowel preparation quality, complete recording of the anatomy, and a 360-degree inspection of the mucosa on withdrawal of the device. A ‘high-quality’ examination was defined as complete visualisation of the luminal mucosa from the dentate line up to and including identification of the rectosigmoid junction.

National and international bowel cancer charities were invited to comment on the study design before the start of the trial. An abstract describing the study was published on the website of the manufacturer SurgEase Innovations,^13^ and updates of its progress were regularly posted on social media including LinkedIn and Twitter.

### Patients

Patients were recruited from general practice or from urgent suspected colorectal cancer referral waiting lists held in secondary care. The rationale for accepting patients from both routes was to increase the capture of patients who needed urgent assessment for their symptoms but faced lengthy delays for face-to-face assessment owing to the pandemic. Adults aged ≥18 years meeting any of the following criteria were included: symptoms fulfilling the 2WW criteria for suspected colorectal cancer;^12^ known patients with IBD experiencing flare symptoms; patients with a suspected new diagnosis of IBD; patients with a positive faecal calprotectin, a raised faecal occult blood (FoB), or faecal immunochemical test (FIT) estimation; or any GI symptoms with a past history of colorectal polyps. Exclusion criteria included the following: the inability to provide informed consent or communicate effectively in English (to limit the number of people in small clinical rooms during the pandemic); pregnancy; allergy to plastics; the presence of an anal stricture; or the inability to take bowel preparation or adopt the left lateral position required for rectal examination. All suitable patients on 2WW lists in secondary care were contacted and could enrol in the study if they agreed. In primary care, clinicians could contact appropriate patients from their clinic lists. Patients were approached either by telephone or face-to-face a week before the examination visit, allowing sufficient time for participants to read the patient information sheet and provide informed consent. Patients were provided with a glycerine suppository to administer at home before attending for examination.

**Figure 2.**
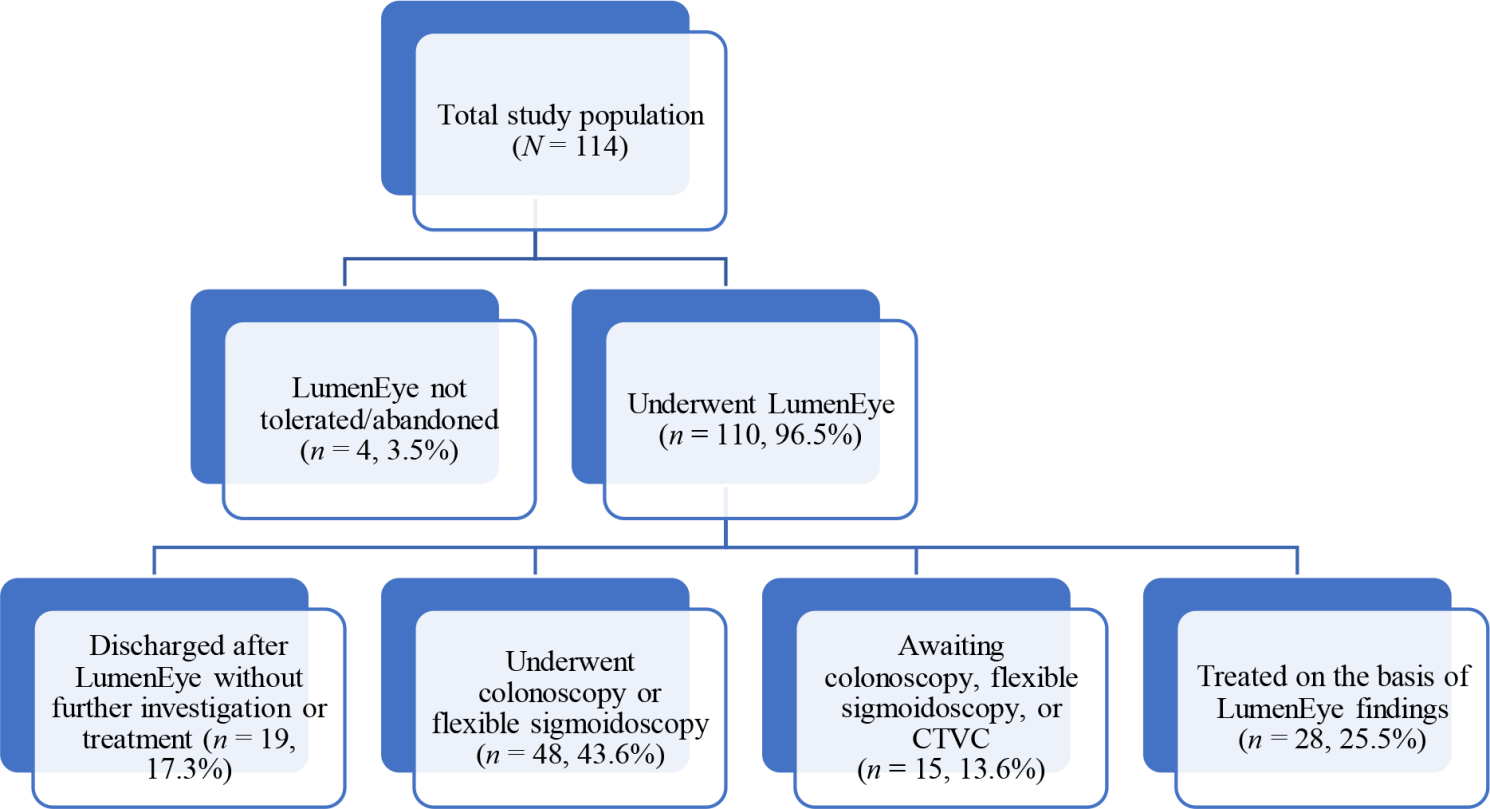
Flow diagram demonstrating the outcome of patients in the trial. Follow-up data are available for 110 of 114 patients. Three patients were unable to tolerate the procedure and one patient was lost to follow-up despite numerous efforts to contact him. CTVC = computed tomography virtual colonoscopy.

Each site was provisionally set a limit of 25 patients. However, owing to factors such as disparate patient footfall, medical personnel availability, and time taken to get relevant approvals, the range of patients recruited by site ranged from 10–34. Following national lockdown restrictions in January 2021, recruitment was paused for 3 months, and permission to extend the trial was granted by the funder and ethics review board. All patients were referred for further investigation at the primary care clinician’s discretion. Patients recruited from the 2WW referral pathway had the diagnostic tests they would have undergone had the LumenEye examination not been performed. The reports of any subsequent investigations were obtained from the patient’s electronic health record. Where appropriate, treatments for haemorrhoids, anal fissures, or IBD could be initiated based on the LumenEye findings in primary care. The results were reported in accordance with the Standards for Reporting of Diagnostic Accuracy (STARD) (Figure 2).

### Tele-endoscopy platform (CHiP)

All clinicians were given training on the CHiP software (Figure 1B and C) and were allocated secure login credentials and passwords. Data were stored on a fully encrypted Azure cloud server, which adhered to all NHS security and governance standards including cyber security, data processing, information management, and penetration testing. The cloud server was hosted by the company that held ISO-27001, Cyber Security Essentials Plus, and NHS Data Security and Protection (DSP) Toolkit accreditation. The decision to use the CHiP platform was left to the primary clinician‘s discretion with simultaneous technical assistance, if needed. A secondary care clinician in the local hospital was available at the time of the examination with simultaneous access to the CHiP platform owing to the scheduling feature on CHiP. Specialists could also dial-in ad hoc, from their laptops or mobile phones, if needed. Videos and/or images were stored and reviewed retrospectively within one working day by an independent expert endoscopist for every patient recruited to the study to minimise the risk of missed pathology. Where pathology was missed by the examining primary care physician, the clinicians and patient were alerted and appropriate action taken.

### Digital rectoscopy

The digital rectoscopy examination was performed with the patient in the left lateral position without sedation using the standard technique for rigid sigmoidoscopy. Manual air insufflation using the in-built bellows is required to achieve optimal rectal distension. Quality assessment of bowel preparation was made by each clinician using criteria based on the Boston Bowel Preparation Scale.^14^ Each bowel was assessed to be good (>80% mucosa visible), average (>50% mucosa visible), or poor (<30% mucosa visible). The duration of the examination and any discomfort experienced by the patient was recorded. Following the examination, a questionnaire developed from a published rigid sigmoidoscopy experience study^15^ was completed by the patient (see Supplementary Appendix S1). A case report form after each examination was completed recording details on bowel preparation, duration of the examination, distance of insertion, pathology detected, number of air insufflations required, and a rating of the ease of use of the LumenEye and CHiP technologies (see Supplementary Appendix S2).

### Outcome measures

The primary outcome was patient and clinician acceptability. Secondary outcomes included safety and analysis of diagnostic accuracy, referral outcomes, and cost.

### Variables

The main independent variables were qualitative assessment of patient comfort, anxiety, convenience, and level of reassurance of having digital rectoscopy assessment in the community. To avoid the risk of reporting bias, the entire cohort of patients was used in the user-feedback analysis.

For clinicians, the level of diagnostic confidence was the main variable. The procedures were performed by 10 primary care practitioners (eight GPs and two advanced nurse practitioners), with variable endoscopy experience ranging from novices to Joint Advisory Group accreditation for flexible endoscopy, which likely influenced clinician confidence and diagnostic ability. A confounding variable was the fact that patients were selected by clinicians, which could positively bias patient responses. There is also likely to be a high pick-up rate of pathology owing to all patients selected having fulfilled either 2WW criteria or having results indicative of having disease. Additionally, a degree of patient response bias is anticipated as they were asked to complete the questionnaire straight after the procedure. Ideally, an independent body would have requested patient feedback separate to the clinical event.

### Statistical analysis

The conditions set by the funding body for this study stipulated trial completion to be achieved within 6 months of the first recruitment. After consultation with each primary care site and with consideration of the risk of national lockdown during the study, a pragmatic monthly recruitment target of one patient per week per site was deemed practical and not burdensome to primary care during times when face-to-face consultations were limited. With 1 month dedicated to training and recruiting NHS sites, a recruitment rate of four patients per month per site was set. A 10% attrition rate was calculated, setting the final recruitment target of 110 over 5 months. Because this study was designed to assess feasibility with qualitative end-points and determine the practicalities of trial delivery for a subsequent phase III study, this approach was deemed appropriate and of similar size to a comparable investigational point-of-care technology study.^16^ Analysis of quantitative data were performed on an as-treated basis and on an intention-to-treat basis in the qualitative domains. Variables were expressed as median and interquartile range (IQR), and the χ^2^ and Kruskal-Wallis tests were used to compare categorical non-parametric data between groups. The Pearson correlation coefficient was used to identify significant relationships between variables. The sensitivity, specificity, positive predictive value (PPV), and negative predictive value (NPV) of the LumenEye against the gold standard (either CTVC, flexible sigmoidoscopy, or colonoscopy) was compared with a receiver operating characteristic (ROC) analysis. All statistical calculations were undertaken using Statistical Package for the Social Sciences (SPSS; version 27). A *P*-value of <0·05 was taken to be statistically significant.

## Results

### Patients

One hundred and fourteen patients were enrolled in the study from five NHS regions between November 2020 and June 2021. Of 114 patients enrolled, three were unable to tolerate the procedure owing to discomfort, and one examination was abandoned by the examining GP owing to the presence of prolapsing haemorrhoids, giving a failure rate of 3.5%. All the failures were males aged 30 years, 36 years, 56 years, and 67 years. On subsequent examination, one had an acute anal fissure, one had a colonic adenomatous polyp, and one had a large prolapsing haemorrhoid. The fourth patient opted not to undergo further investigation. A total of 110 examinations were completed and formed the denominator for the analysis of the LumenEye diagnostic performance.

The median age of the patients was 53 years (IQR 36–67) and 64 (58.2%) were male (Table 1). Reported presenting complaints in diminishing order of frequency were rectal bleeding (*n* = 72, 65.5%), change in bowel habit (*n* = 59, 53.6%), proctalgia (*n* = 30, 27.3%), symptoms of rectal mucosal prolapse suggestive of haemorrhoids (*n* = 25, 22.7%), and mucous discharge (*n* = 19, 17.3%) (Table 1). Four (3.5%) of the 114 recruited patients had an established diagnosis of IBD and 13 (11.8%) had a family history of bowel cancer.

**Table 1.** Demographics and presenting symptoms of participants (*N* = 110)

**Category**	Variable	*n*	%
Sex	Female	46	41.8
	Male	64	58.2
Diagnosis of IBD	Yes	4	3.6
	No	106	96.4
Rectal bleeding	Yes	72	65.5
	No	38	34.5
Change in bowel habit	Yes	59	53.6
	No	51	46.4
Mucus discharge	Yes	19	17.3
	No	91	82.7
Proctalgia	Yes	30	27.3
	No	80	72.7
Pruritus ani	Yes	10	9.1
	No	100	90.9
Haemorrhoids	Yes	25	22.7
	No	85	77.3
Mass or prolapse	Yes	7	6.4
	No	103	93.6
Family history of bowel cancer	Yes	13	11.8
	No	97	88.2

IBD = irritable bowel disease.

Nineteen (17.3%) of the 110 examined patients were discharged without further investigation or treatment after LumenEye, 48 (43.6%) had formal investigation (a colonoscopy, flexible sigmoidoscopy, or CTVC), 15 (13.6%) were still awaiting these procedures or the results of their investigation were not available at the time of analysis, and 28 (25.5%) were treated based on the LumenEye findings (Figure 2).

### Patient acceptability

Questionnaires were returned by 111 patients including three patients with an incomplete examination (see Supplementary Table S1). Completion of forms was unsupervised to reduce staff contact and COVID-19 risk.

The examination was more comfortable than expected in three-quarters (*n* = 82/110, 74.5%), although one-quarter (*n* = 28/106, 26.4%) experienced some degree of discomfort as indicated by the visual analogue scale. Twenty-four of 110 patients (21.8%) had previously undergone a rigid sigmoidoscopy procedure and the majority (*n* = 16/24, 66.7%) found the LumenEye examination to be ‘better’ or ‘much better’ than their previous experience. Just over half of the participants (*n* = 60/111, 54.1%) did not report feelings of embarrassment and almost all (*n* = 106/111, 95.5%) felt they had sufficient privacy in the community setting during the intervention. Moreover, the community setting was reported to be the most convenient (*n* = 104/110, 94.5%) compared with a hospital and the preferred setting for future examinations, as reported by 76 (70.4%) of 108 responders. The digital sharing of images over the CHiP platform and discussion of clinical cases with specialists was felt to be beneficial by 103 (92.8%) of 111 responders, while 106 (96.4%) of 110 responders were comfortable with this type of interaction. Almost all participants (*n* = 108/110, 98.2%) felt reassured their images were being reviewed by a specialist.

### Clinician acceptability

Clinicians reported a high rate of confidence in the quality of their assessment of the rectum (see Supplementary Table S1). They were ‘very confident’ in 62 (54%) or ‘reasonably confident’ in 38 (33%) of the 114 responses. Clinicians lacking endoscopy experience stated that anatomy recognition and anorectal disease identification required additional training.

### Quality of the examination

Overall, 109 patients used a glycerine suppository and one used a rectal irrigation system, which she routinely used. A repeat suppository was required in four (3.6%) patients owing to inadequate views. Bowel preparation was stated to be ‘good’ in 75 (68.2%), ‘average’ in 29 (26.4%), and ‘poor’ in six (5.5%) examinations (data not shown).

The median distance of insertion of the LumenEye was 15 cm (IQR 15.0–17.5). A high-quality examination was achieved in 98 (89.1%) of the 110 patients, with visualisation of the upper rectum being achieved in 109 (95.6%) irrespective of sex (*P* = 0.424). The rectosigmoid junction was not visualised in 16 (14.0%) of patients, and therefore deemed to be of low quality (data not shown).

The median number of air insufflations using the bellows of the instrument was 8 (IQR 6–12). This significantly correlated with the quality of the examination; the rectosigmoid junction was visualised in 97.8% of patients (*n* = 46/47) who had >8 insufflations compared with 78% in those who had ≤8 (*P* = 0.002). The median duration of the examination in primary care was 5 minutes (IQR 4–8) with no statistical correlation between duration and quality (*P* = 0.646) (data not shown).

### Diagnostic sensitivity and specificity of the LumenEye

The abnormalities found on LumenEye examination are shown in Table 2. A significant finding (cancer, polyp, or inflammation) was found in 24 (21.8%) of 110 patients, two of whom had a cancer. Forty-eight (43.6%) of the 110 patients were referred for colonoscopy, flexible sigmoidoscopy, or CTVC, and among these the sensitivity and specificity of the LumenEye in identifying any pathology were 90.0% and 88.9%, respectively. For the two patients found to have a cancer, the sensitivity and specificity of the LumenEye were both 100%, for polyps 83.3% and 97.8%, and for inflammation 100% and 93.2%. Five abnormalities (four polyps and one mild erythema) were missed by the examining primary care physician. An additional independent expert endoscopist reviewed the recorded images and videos from these patients. Four out of the five pathologies missed were seen on the recordings giving a true miss rate for the LumenEye of 0.9% (*n* = 1/110).

**Table 2.** Significant abnormality, defined as polyps, inflammation, or cancer, identified by the primary care physician at the time of the LumenEye examination (*N* = 110 patients), compared with the findings of follow-up investigation or expert review, and sensitivity, specificity, positive predictive value, and negative predictive value of the LumenEye in the identification of pathology compared with subsequent colonoscopy, flexible sigmoidoscopy, or CTVC findings

Pathology recorded, *n*		Cancer	Polyp	Inflammation
Identified in primary care		2	6	16
Identified after formal investigation or expert review of recorded images		2	11	15
**All patients**	**Any pathology**	**Cancer**	**Polyp**	**Inflammation**
TP, *n*	9	2	5	4
TN, *n*	32	46	45	41
FP, *n*	4	0	1	3
FN, *n*	1	0	1	0
Sensitivity = TP/TP + FN, %	90.0	100	83.3	100
Specificity = TN/TN + FP, %	88.9	100	97.8	93.2
PPV = TP/(TP + FP), %	69.2	100	83.3	57.1
NPV = TN/(TN + FN), %	97.0	100	97.8	100
**Patients presenting with rectal bleeding (** ***n*** **= 28)**	**Any pathology**	**Cancer**	**Polyp**	**Inflammation**
TP, *n*	8	2	3	4
TN, *n*	15	25	24	21
FP, *n*	4	0	1	3
FN, *n*	1	0	1	0
Sensitivity = TP/TP + FN, %	88.9	100	75.0	100
Specificity = TN/TN + FP, %	78.9	100	96.0	87.5
PPV = TP/TP + FP, %	66.7	100	75.0	57.1
NPV = TN/(TN + FN), %	93.8	100	96.0	100

CTVC = computed tomography virtual colonoscopy. FN = false negative. FP = false positive. NPV = negative predictive value. PPV = positive predictive value. TN = true negative. TP = true positive.

The performance of the LumenEye was analysed in a cohort of 28 patients whose presenting symptom was rectal bleeding and who subsequently had formal investigation (Table 2). In this group, the sensitivity and specificity of the LumenEye in detecting any pathology were 88.9% and 78.9%. For cancer they were both 100%, for polyps 75.0% and 96.0%, and for inflammation 100% and 87.5%. An ROC analysis demonstrated an area under the curve (AUC) diagnostic accuracy for any pathology of 81.9% (95% CI = 66.2 to 97.6%) for the LumenEye compared with formal investigation in the 48 patients who had both examinations.

A power calculation was performed to plan future trials. Using data of patients with lower GI symptoms examined by the LumenEye who subsequently had formal investigation, assuming an alpha cut-off of 95% (alpha being the probability of a type I error, and falsely rejecting the null hypothesis = 0.05), to detect any pathology with the LumenEye (polyp, cancer, or inflammation), 143 patients would be required in each arm to demonstrate non-inferiority to flexible endoscopy or CTVC. To achieve non-inferiority in polyp detection, 7216 patients would be needed with 3608 in each group.

### The CHiP platform

The CHiP platform provided specialist real-time second opinion in 26 (23.6%) of the 110 examinations. The median number of attempts to establish a connection was one. There was no delay or lag to the connection reported in 20 (76.9%) of 26 patients whose rectoscopy was accompanied by an available connection. Of the 26 uses of the CHiP system, 23 (88.5%) were judged to be ‘excellent’ with the options of ‘good’, ‘average’, or ‘poor’ scoring 3.8% in each category. In two study centres with no Wi-Fi availability a tethered mobile phone device was used, but the poor experience in these centres demonstrates the importance of a reliable internet connection (data not shown).

### Cost

In total, 19 (17.3%) patients were discharged from further care after the LumenEye procedure (Figure 2), who would otherwise have been referred to secondary care. Further investigation and cost would have included an outpatient department (OPD) assessment (cost 157 GBP) and investigation (colonoscopy 478 GBP, flexible sigmoidoscopy 322 GBP, and CT scan 95 GBP).^17^ The LumenEye intervention costs between 30 and 50 GBP per procedure. On the assumption that each patient would have had an OPD assessment followed by colonoscopy and by averaging the cost of LumenEye to 40 GBP, on direct cost comparison of the interventions, the LumenEye pathway would yield a net saving of 11 305 GBP. Nine patients awaiting further investigation did not have any booked investigation at the time of analysis, demonstrating a potential patient discharge rate of 25% after the LumenEye intervention.

## Discussion

### Summary

This phase I prospective pilot study has demonstrated feasibility and high levels of patient acceptability of the LumenEye rectoscope, and that it is possible to train primary care practitioners to safely perform digital rectoscopy in the community. Moreover, it was possible to introduce a quality-performance assessment suitable for clinical governance, which would further support its wider adoption. Digital rectoscopy by LumenEye in primary care therefore has the potential to reduce the burden on secondary referral pathways and endoscopy services without compromising patient safety.

LumenEye is safe and well accepted by patients and clinicians for rectal examination in primary care, with good diagnostic accuracy. Digital rectoscopy can rationalise and expedite referral from primary to secondary care and reduce the rate of unnecessary referrals avoiding the cost of investigation and clinician time. Digital rectoscopy has an acceptable diagnostic performance in the rectum when compared with flexible endoscopy, with an overall diagnostic sensitivity, specificity, and negative and positive predictive values of 90.0%, 88.9%, 97.0%, and 69.2%, respectively. Only 3.5% of procedures were abandoned. Pathology that required further secondary care input was identified by the GP in 24 (21.8%) patients. The primary care physicians involved were not provided with formal training on pathology recognition, which may explain the positive predictive value of only 69.2%. This would be improved with a formal training programme as identified by clinicians taking part in this study.

### Strengths and limitations

Almost half of patients (43%) in this cohort avoided referral to secondary care or were treated in primary care based on their examination in primary care (see Figure 2). This is likely to be an underestimation of the potential deferral rate as patients who fulfilled the 2WW criteria were included, which mandated a referral to secondary care. In the future, stratifying patients according to risk profile, symptomology and safeguarding the LumenEye assessment with a FIT^18^ test will further improve the diagnostic utility of this device and avoid referrals in many more patients. This could make a huge contribution to the workload of gastroenterology and colorectal services, and diminish the cost of referral and patient anxiety.

The LumenEye system has several advantages over flexible sigmoidoscopy systems, with low maintenance and sterilisation costs (5 GBP per procedure) and being deployable in any general practice setting. Where there is diagnostic doubt over encountered pathology, the associated CHiP software can link the primary care physician immediately to an expert secondary opinion.

This study has some obvious limitations. The sample of 110 patients is small, and the recruitment process may have introduced selection bias. The LumenEye examination is limited to the rectosigmoid and cannot directly identify more proximal pathology. Furthermore, accessibility to an immediate specialist opinion via CHiP is dependent on internet access. However, where internet access is limited, LumenEye can be used in ‘offline mode’. This enables videos and images to still be stored locally on the machine, with deferred upload of assets onto the cloud once internet access is re-established.

### Comparison with existing literature

Endoscopy services in the UK are currently overwhelmed.^19^ Patients are needlessly waiting longer than 2 weeks for specialist review^5^ and 97% of patients referred through the 2WW pathway do not have cancer, consuming valuable NHS resources while causing anxiety to patients.^10^ These pressures have only been exacerbated by COVID-19, which has further reduced capacity^6^ and increased waiting times.^7^ To the authors' knowledge this is the first feasibility study trialling the use of digital rectoscopy in the community. In the present study, nearly one-quarter of the 110 patients examined did not require referral to secondary care. If implemented on a national scale, this technology could avoid 135 000 endoscopies in the UK, amounting to a saving of 75 million GBP annually in endoscopy costs alone.^20^ Digital rectoscopy offers a potential solution to helping to relieve the endoscopy burden. Radical changes to current referral pathways are now required, similar to what has been achieved with teledermatology in primary care.^21^

### Implications for research

Primary care physicians need further training in pathology identification; although, this analysis has established the statistical power required for a further trial in primary care. A phase III randomised control trial of the use of digital rectoscopy in primary care is now planned based on the statistical power calculation deduced from this study.

## Data

Anonymised patient level data is available from the authors on reasonable request.
